# Comparison of gene mutations of liver metastases to the primary tumor in colorectal cancer: lessons from a small cohort

**DOI:** 10.3389/fonc.2026.1924092

**Published:** 2026-09-15

**Authors:** Erzsebet Rásó, Tamas Barbai, Laura Vizkeleti, Andrea Uhlyarik, József Tímár

**Affiliations:** 1 Department of Pathology, Forensic and Insurance Medicine, Semmelweis University, Budapest, Hungary; 2 Institute of Molecular Life Sciences, HUN-REN Research Center for Natural Sciences, Budapest, Hungary; 3 Institute of Pancreatic Diseases, Semmelweis University, Budapest, Hungary

**Keywords:** colorectal cancer, liver metastasis, mutational profile, NGS, primary tumor

## Abstract

**Introduction:**

Upfront molecular diagnostics are used to characterize genetic features of colorectal cancer, guiding therapy. Since colorectal cancer is usually surgically resected, these samples are used for such characterizations. The extent to which liver metastases maintain the genetic profile of the primary tumor is a key question.

**Methods:**

Here, we used a small six-case pretreatment colorectal cancer cohort where targeted next-generation sequencing (NGS) was employed to compare liver metastases to the primary tumors.

**Results:**

We found, similar to previous reports, that the major genetic drivers are maintained in the liver metastases; however, changes at this level can still be detected. We also observed that in the majority of cases, the genetic profile of the primary tumor is partially or completely lost in liver metastases. On the other hand, we detected the metastasis-selective emergence of ABL1, HER2, RET, KIT, KDR, and VHL mutations.

**Discussion:**

Although our study is only hypothesis-generating, these data support the view that genetic analysis of colorectal cancer liver metastases may identify novel alterations compared to the primary tumors, which can be clinically exploited during disease management.

## Introduction

The liver is one of the most frequently involved organs in the metastatic dissemination of cancers. Due to the anatomy, the liver serves as the first filter organ for the hematogenous dissemination of gastrointestinal-tract cancers (portal vein drainage). However, this fact is only one of the necessary conditions for the successful liver colonization of cancers. On one hand, the liver must provide a supportive microenvironment for colonizing cancer cells; on the other hand, cancer cells must be able to establish micrometastases. For this, cancer cells must rely on genetic capabilities, such as “stemness” and proliferative and anti-apoptotic strategies (1, 2).

For a long time, it was considered evident that metastases (including those in the liver) are genetically very similar, if not identical, to the primary tumor as far as the driver oncogenes are concerned. Later, this idea was redefined, recognizing that the primary tumor is genetically heterogeneous and only some of its subclones possess the ability to colonize the liver. Large-scale genomic analysis of colorectal cancer with liver metastasis has documented that the major oncogenes and suppressor genes are present in liver metastases, including KRAS, NRAS, BRAF, and PIK3CA, as well as P53, SMAD4, and APC (3, 4). Meanwhile, there are five distinct potential genetic scenarios for tumor progression, including liver metastasis: linear clonal evolution, parallel evolution, clonal elimination, return of the same clone, and branching evolution (5, 6). Each of these progression forms has different consequences for the genetic composition of the metastasis compared to the primary tumor. Accordingly, based on the primary tumor, it is impossible to predict the genetic composition of the (liver) metastasis; only direct evaluation can reveal the unique genetic features that may influence or determine clinical decisions and therapeutic strategies. In that respect, it was found that the consensus molecular subtype profile of the metastases can differ significantly from their paired primary (7). Comparative systematic studies on colorectal cancer liver metastases identified their clonal progression from the primary tumor (8). Based on relatively small paired cohorts, it was observed that the proportion of metastasis-specific gene alterations is relatively low: 19% (3). However, another study detected a wide range of shared genetic alterations in individual cases, from low (16.8%) to a very high proportion (86.8%) (9). Accordingly, in this small cohort, we tested colorectal liver metastases for clinically relevant genetic alterations compared to their primary tumors, since even today, the primary tumor is the source of information for clinical decision-making.

## Materials and methods

### Patients

In a previous retrospective study, we collected clinical data of colorectal cancer patients (all adenocarcinomas) treated at the Hungarian Defence Forces Medical Center. The analysis was approved by the Institutional Review Board of Semmelweis University (19/1043). We collected formalin-fixed paraffin-embedded (FFPE) tissue of pre-treatment samples from 10 liver metastatic patients who underwent surgery for their metastasis, and we also collected the matched FFPE blocks of the surgically removed primary tumors in six cases. Initial molecular diagnostics included KRAS exon 2 mutation testing (10) according to the then-existing guidelines and mismatch repair (MMR) protein analysis by immunohistochemistry (11). Unfortunately, the other four primary samples were not appropriate for next-generation sequencing (NGS) analysis due to quality reasons. The clinical data of the patients are shown in **Table 1**.

**Table 1:** Patient cohort.

Case no.	Sex	Age	Location of the primary tumor	Stage	Grade	Liver metastasis	MMR	Treatment
1	Male	74	Sigmoideal	T3N0	2	S	p	BEV-FOLFIRI
2	Male	69	Flexura hepatica	T3N2	3	S	p	BEV-FOLFIRI
3	Male	49	Left colon	T3N1	3	M	p	CET-FOLFIRI
4	Female	61	Rectal	T3N2	2	S	p	BEV-FOLFIRI
5	Male	75	Rectosigmoideal	T3N1	2	M	p	CET-FOLFIRI
6	Male	60	Cecal	T3N1	1	S	p	BEV-FOLFIRI
7	Male	72	Rectosigmoideal	T3N2	2	S	p	BEV-FOLFIRI
8	Male	78	Cecal	T4N0	2	S	d	FOLFIRI
9	Male	66	Left colon	T3N1	1	S	d	BEV-FOLFIRI
10	Male	57	Transversal colon	T4N0	2	S	p	BEV-FOLFIRI

BEV, bevacizumab; CET, cetuximab; FOLFIRI, leucovorin; 5-FU, irinotecan; M, metachronous; MMR, mismatch repair; p, proficient; d, deficient; S, synchronous.

### Next-generation sequencing

Samples were assessed by light microscopy, and tumor-rich areas (T/N ratio >50%) were macrodissected. HighPur PCR Template Kit (Roche, Mannheim, Germany) was then used to isolate genomic DNA. Quantity and purity were assessed with a Qubit 4 Fluorometer using the Qubit dsDNA HS Assay Kit (Thermo Fisher Scientific, Paisley, UK).

Library preparation was performed with the Ion AmpliSeq Cancer Hotspot Panel v2 (Thermo Fisher Scientific) of 50 genes (ABL1, EGFR, GNAS, KRAS, PTPN11, AKT1, ERBB2, GNAQ, MET, RB1, ALK, ERBB4, HNF1A, MLH1, RET, APC, EZH2, HRAS, MPL, SMAD4, ATM, FBXW7, IDH1, NOTCH1, SMARCB1, BRAF, FGFR1, JAK2, NPM1, SMO, CDH1, FGFR2, JAK3, NRAS, SRC, CDKN2A, FGFR3, IDH2, PDGFRA, STK11, CSF1R, FLT3, KDR, PIK3CA, TP53, CTNNB1, GNA11, KIT, PTEN, and VHL). First, 10 ng of genomic DNA was subjected to multiplex PCR amplification using gene-specific primer pools, followed by enzymatic digestion and ligation of Ion Xpress Barcode Adapters. Barcoded libraries were quantified with the Ion Library TaqMan Quantitation Kit and processed on the Ion Chef System (Thermo Fisher Scientific) for automated template preparation and chip loading.

Sequencing was carried out on the Ion PGM Sequencing System using Ion 318 chips, following the manufacturer’s protocol, with 5% VAF sensitivity and an average sequencing depth of 200×. Primary data processing, including base calling, alignment, and variant calling, was performed using Ion Torrent Suite Software. Variant annotation and filtering were conducted in Ion Reporter Software (v5.20) using the NCBI ClinVar and the Franklin (Qiagen, Hilden, Germany) databases. The raw sequencing data for six paired and four liver metastasis cases are shown in **Supplementary Tables S1**-**S6**, corresponding to the case numbers in **Table 1**, where “STa” denotes primary samples and “STb” denotes liver metastatic samples.

### NGS validation cohort

The Cancer Genome Atlas colorectal adenocarcinoma database cohort (12) and two Memorial Sloan Kettering Cancer Center colorectal cancer cohorts (13, 14) of 69 liver metastases and their primary pairs were analyzed for 35 selected genes using the cBioPortal for Cancer Genomics online tool to visualize mutation frequency and type in each sample.

## Results

With a 15% variant allele frequency (VAF) cut-off, NGS detected mutations in 24 genes in primary tumors and 29 genes in liver metastases. Note that none of the mutations had an allele frequency >50% VAF. We did not detect mutations in PTPN11, HNF1A, MPL, SMARCB1, JAK2/3, NPM, SMO, CDH1, SRC, CSF1, ALK, FLT3, or CTNNB1 genes in this small cohort. The frequency of gene mutations in primary tumor versus liver metastasis is shown in **Figure 1**. In both primary tumors and liver metastases, the most frequent mutations were found in suppressor genes, P53, RB1, PTEN, and APC, and in the KRAS oncogene (exon 3/4). Notably, HRAS and BRAF mutations were found in liver metastases. SMAD4 and PIK3CA driver mutations were similarly prevalent in both primary and metastatic tumors. We also observed that the MET mutation was present exclusively in primary tumors. When we looked for liver metastasis-selective gene alterations, we detected HER2, ERBB4, RET, KIT, ABL1, and VHL mutations (**Figure 1**). We compared the incidence of gene mutations in our cohort to the TCGA/MSKC liver metastatic colorectal cancer dataset, which is based on 69 liver metastatic cases. This comparison indicates that KRAS and P53 mutation frequencies are similar to our cohort, but the majority of mutations are more prevalent in our small cohort (**Table 2**).

**Figure 1:**
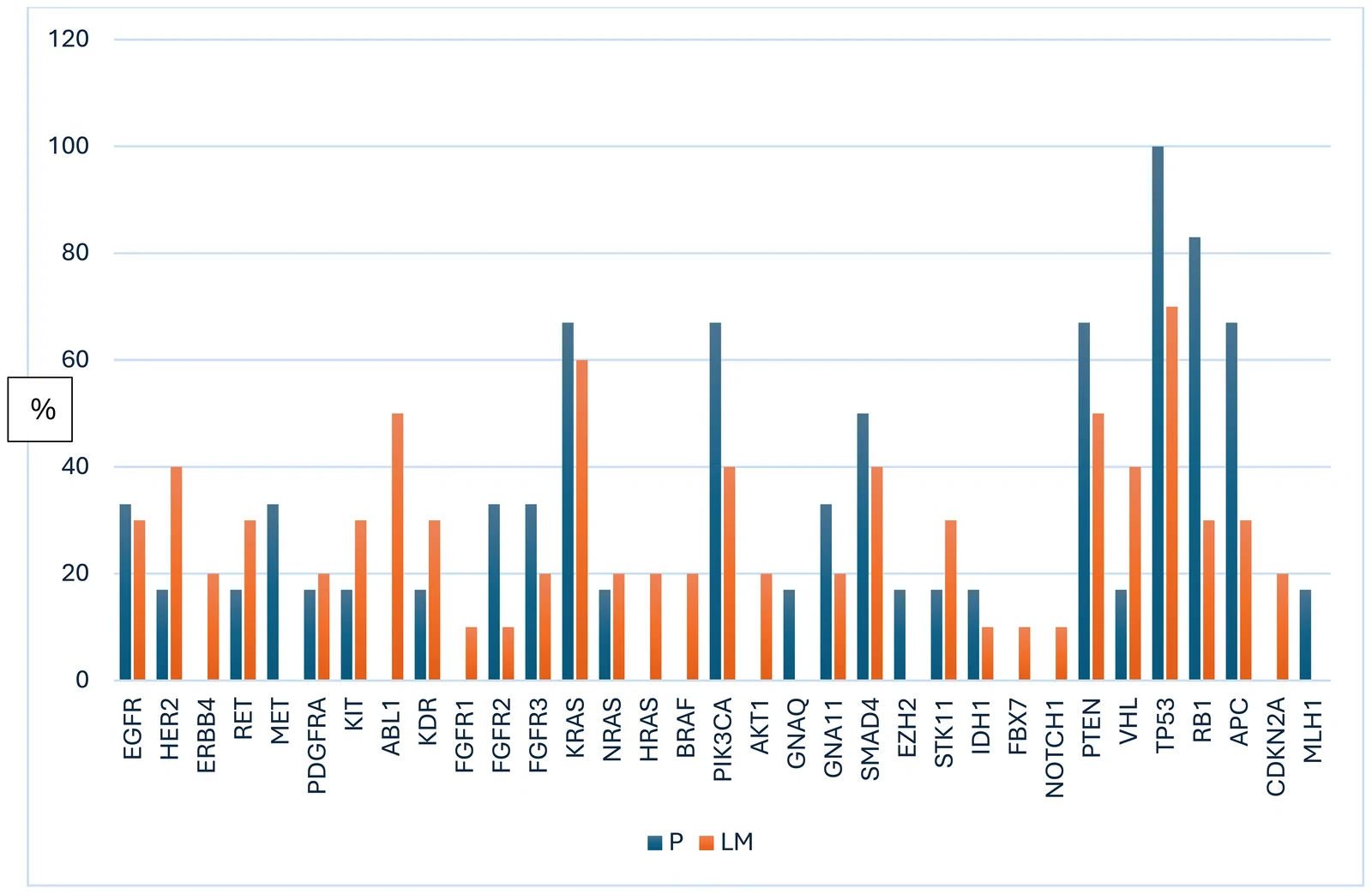
Mutational profiles of the primary tumors versus liver metastases. Data are expressed in %. P, primary tumor and LM, liver metastasis.

**Table 2:** Comparison of mutation patterns of the primary colorectal cancers to liver metastases using *in silico* data of TCGA+MSKC.

Mutant gene frequency	Primary (n=69)	Liver metastasis (n=69)
	%	%
EGFR	3	1
HER2	4	3
ERBB4	8	3
RET	5	0
MET	3	1
PDGFRA	4	4
KIT	4	0
ABL1	3	0
KDR	5	1
FGFR1	2	0
FGFR2	2	0
FGFR3	3	1
ALK	7	1
FLT3	2	1
KRAS	38	57
NRAS	6	3
HRAS	1	0
BRAF	14	4
PIK3CA	23	20
AKT1	2	1
GNAQ	1	0
GNA11	1	0
SMAD4	12	16
EZH2	0	0
STK11	1	0
IDH1	1	0
FBX7	1	0
NOTCH1	6	1
PTEN	6	3
VHL	1	0
TP53	55	83
RB1	3	0
APC	69	90
CDKN2A	1	0

Next, we compared the genetic profile of the primary tumor to its corresponding liver metastasis to assess the extent of profile maintenance (**Figure 2**). In total, we detected 52 mutations (~9/sample) in primary tumors. We found that in two cases (P1 and P4), the primary profile was preserved in liver metastases, in two other cases (P3 and P5), it was almost completely lost, while in another two cases (P2 and P6), it was significantly altered (**Figure 2**).

**Figure 2:**
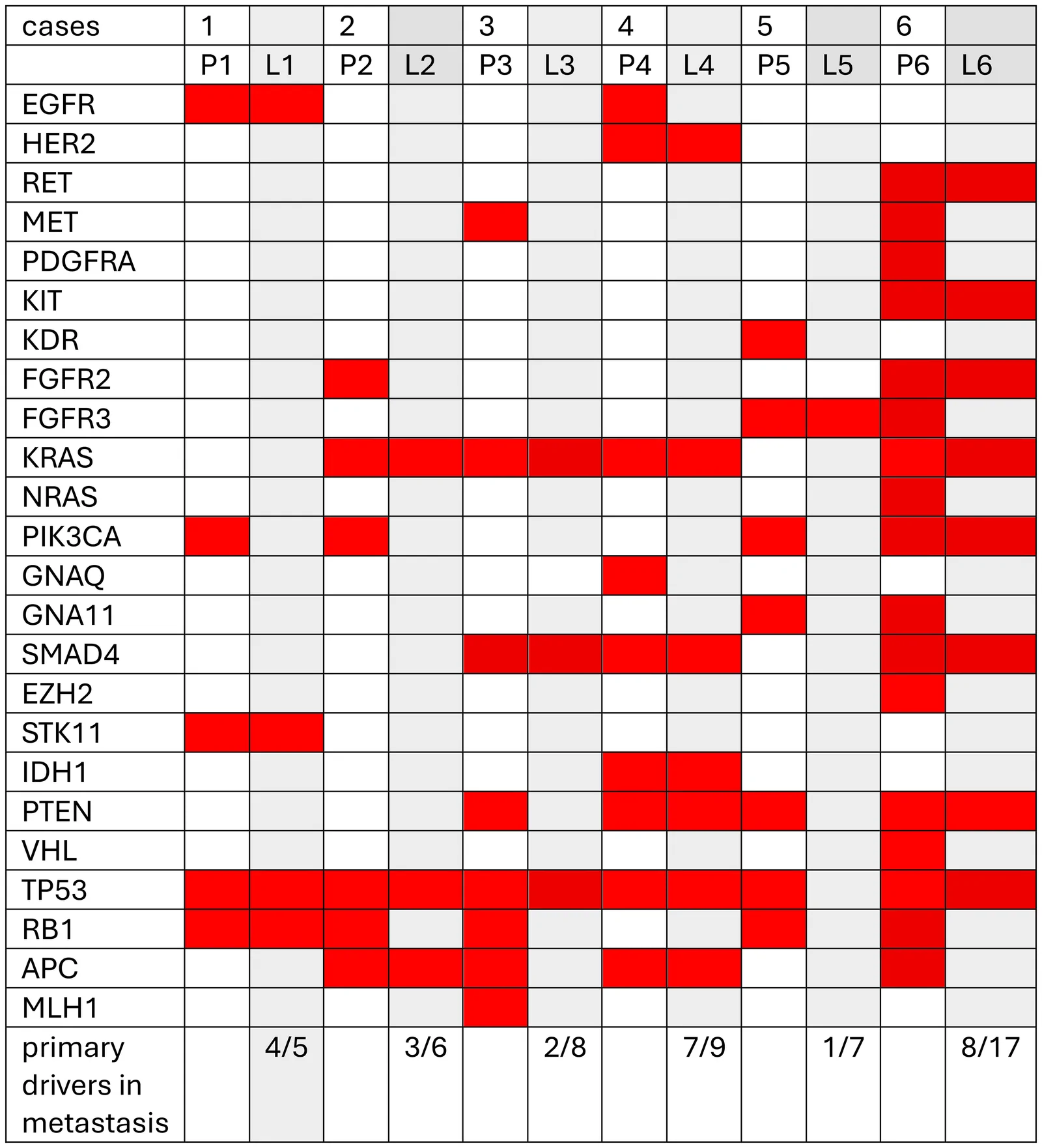
Preservation of the mutation profile of the primary tumor in the corresponding liver metastasis.

Next, we examined liver-specific driver patterns against the total mutations (**Figure 3**). In total, we detected 56 mutations (~8/sample), a rate similar to that in primary tumors. It is interesting, however, that the majority of mutations were unique to liver metastatic samples (30/56). The analysis demonstrated cases where novel mutations were predominant (L1 and L3) and cases where these were rare or missing (L2 and L5). Furthermore, in one case (L1), an NRAS mutation emerged, while in L3, a BRAF mutation emerged in liver metastasis. When we analyzed PIK3CA mutations in the primary tumor compared to their metastases, we observed that in three out of four cases where the primary was positive, this mutation was lost in liver metastasis (**Figure 2**). However, in liver metastases, PIK3CA mutations emerged in another two cases where the primary tumor was negative (**Figure 3**). Next, we also compared the four liver metastases for which primary tumor data were missing to the paired ones. This analysis suggested that the mutation patterns of those samples were similar to the paired ones except for case L7 (**Figure 3**).

**Figure 3:**
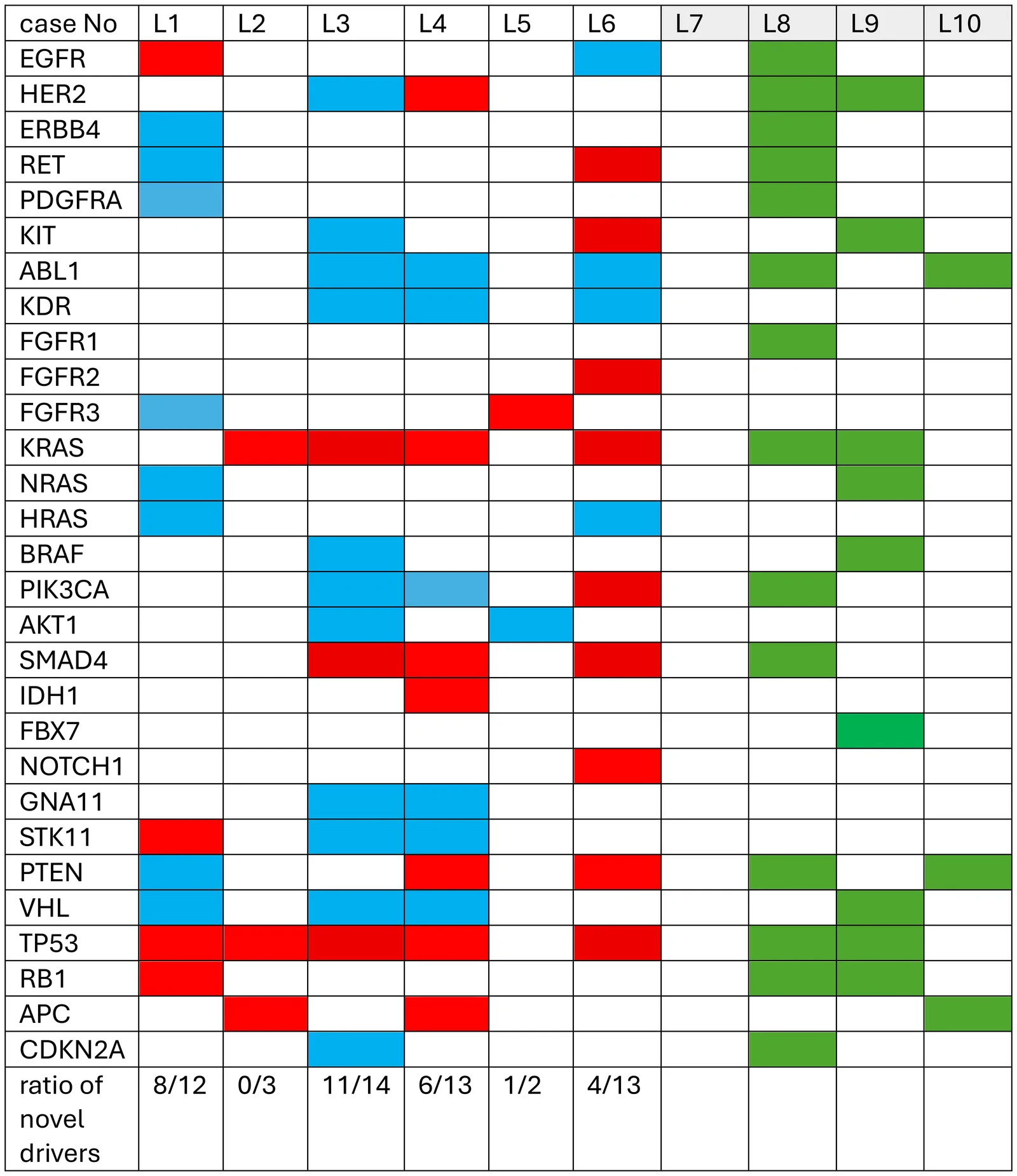
Liver metastasis-specific driver setup in individual cases. Red, primary mutation and blue, mutation exclusive to the liver metastasis. L7–L10, no data on the primary tumor (green).

## Discussion

Our comparative genetic analysis of a small cohort of colorectal cancer primary tumors and their liver metastases, obtained before any treatment, demonstrated that the mutation frequency (of the panel of 50 genes) in the primary tumor compared to the liver metastasis is very similar, supporting previous observations on large case series (3, 4, 8, 15). We observed that major suppressor and oncogenic driver profiles appeared similar in liver metastases compared to the primary tumor. Meanwhile, the pairwise comparison demonstrated that the majority of liver metastasis mutations were specific to them, indicating frequent switching of the primary tumor genetic profile, which is unexpected. However, a MET mutation was found in the primary tumor, whereas HER2, ERBB4, RET, KDR, KIT, and ABL1 were detected more frequently in metastases. These mutations are more frequent in our small cohort than in the TCGA dataset, which can be explained by the fact that these are Hungarian patients of homogeneous ethnicity and/or by the unique nature of this cohort of KRAS exon 2 wild-type cases.

This small case series of liver metastatic colorectal cancer provided data indicating that target sequencing of liver metastasis can offer additional information on the mutational profile of the progressing disease, which can be clinically relevant. On one hand, novel RAS mutations can appear in liver metastases, or KRAS mutations can disappear in metastasis, both of which affect the applicability of anti-EGFR antibody therapies. Such changes are expected to occur in metachronous metastases, but here, we observed the majority in synchronous metastases. Concerning case no. 3, the novel BRAF hotspot mutation of metastasis could have affected the therapy using cetuximab.

Importantly, it was interesting to observe the frequency of novel VHL mutations in liver metastases, with or without KDR or PDGFRA mutations—all predictive markers for anti-VEGF antibody or multikinase angiogenesis inhibitors, further supporting the use of bevacizumab in case nos. 1, 4, and 9. In a previous study on a relatively large cohort of liver metastatic colorectal cancer, overexpression of VEGF-A and ANGP-2 was detected, signaling the upregulation of the hypoxia-signaling pathway (16, 17). The observed frequent liver metastasis-selective VHL mutations may provide a genetic background for this phenomenon. PIK3CA mutations are relatively frequent in colorectal cancer and were also frequent in liver metastases; these are established targets for PIK3CA inhibitors in other cancer types. However, it is interesting that while this mutation was frequently lost in metastases, it appeared in liver metastases that were primarily negative for it, again demonstrating clonal switch during progression. Targeted sequencing of liver metastases also revealed mutations in ABL, RET, KIT, and FGFRs, all well-established targets for specific kinase inhibitors in other cancer types. Using European Society of Medical Oncology ESMO Scale for Actionability of Molecular Targets ranking, they all fall into the IIa and IIIa categories (18). Note that the majority of these “novel” liver metastasis mutations are usually rare in the TCGA-MSKC dataset (**Table 2**), suggesting that re-sequencing liver metastases, even after sequencing of the primary tumor, can be a clinically relevant approach.

Previous studies on colorectal cancer progression to the liver detected unique alterations in metastases compared to the primary tumor, affecting various genes not considered oncogenic or suppressor genes (19, 20). Furthermore, the consensus molecular subtype profiles of colorectal cancer differ in liver metastases compared to primary tumors (21, 22). Our findings from this small cohort are only hypothesis-generating but may prompt more systematic analyses of larger cohorts to clarify these issues.

## Data Availability

The datasets presented in this study can be found in online repositories. The names of the repository/repositories and accession number(s) can be found in the article/**Supplementary Material**.
